# Consistency in the supply of larval fishes among coral reefs in French Polynesia

**DOI:** 10.1371/journal.pone.0178795

**Published:** 2017-06-08

**Authors:** Marc Besson, Camille Gache, Rohan M. Brooker, Rakamaly Madi Moussa, Viliame Pita Waqalevu, Moana LeRohellec, Vincent Jaouen, Kévin Peyrusse, Cécile Berthe, Frédéric Bertucci, Hugo Jacob, Christophe Brié, Bruno Wan, René Galzin, David Lecchini

**Affiliations:** 1 PSL Research University: EPHE-UPVD-CNRS, USR 3278 CRIOBE, BP, Moorea, French Polynesia; 2 UMR 7232, CNRS-UPMC, Observatoire Océanologique de Banyuls-sur-Mer, Banyuls-sur-Mer, France; 3 School Marine Science and Policy, University of Delaware, Lewes, DE, United States of America; 4 School of Marine Studies, Institute of Marine Resources University of the South Pacific, Suva, Fiji; 5 Institute for Pacific Coral Reefs, IRCP, Moorea, French Polynesia; 6 International Atomic Energy Agency, Environment Laboratories (IAEA-EL), Principality of Monaco, Monaco; 7 Tahiti Perles, Papeete, Tahiti, French Polynesia; 8 Laboratoire d'Excellence “CORAIL”, Moorea, French Polynesia; Department of Agriculture and Water Resources, AUSTRALIA

## Abstract

For marine fishes with a bipartite life cycle, pelagic larval dispersal can shape the distribution, connectivity, composition and resilience of adult populations. Numerous studies of larval dispersal, and associated settlement and recruitment processes, have examined the relationship between population connectivity and oceanographic features. However, relatively little is known about spatial and temporal variation in the abundance of larvae settling among different reefs and the extent to which the species assemblage of larvae settling at one location is reflective of the assemblage in neighbouring areas. Here, using crest nets, which provide a non-selective measure of the total abundance and assemblage of larvae settling to a reef (*i*.*e*. larval supply), we collected larval coral reef fishes at five locations surrounding two spatially disparate French Polynesian islands: Moorea and Nengo-Nengo. Overall, larval settlement patterns were correlated with the lunar cycle, with larval abundance peaking during the new moon. Although there were some spatial differences in larval supply among the five monitored sites, settlement patterns were largely consistent, even at the species level, irrespective of factors such as coastline orientation or distance between sites. This study provides further insights into the mechanisms driving patterns of dispersal and settlement of larval fishes over large spatial scales.

## Introduction

Identifying the factors that determine patterns of distribution and abundance is a fundamental goal of ecology and conservation biology [1]. In species that undergo ontogenetic shifts in habitat use, patterns of early life-stage dispersal, return (*i*.*e*. settlement), and persistence in adult habitats (*i*.*e*. recruitment) can impact population dynamics and connectivity [2]. Consequently, deciphering these patterns is essential for understanding population persistence and predicting resilience to environmental change [1,3,4].

The majority of coral reef fishes have a bipartite life cycle, consisting of a pelagic larval stage followed by a largely sedentary reef-associated juvenile/adult stage [3,5]. In these species, the pelagic stage (*i*.*e*. larvae) primarily facilitates dispersal, driven by both physical (*e*.*g*. oceanic currents) and biological (*e*.*g*. directional swimming and sensory perception) processes [4–6]. These processes act at a variety of spatial and temporal scales, with their relevance to dispersal varying depending on larval development and pelagic larval duration. For example, young larvae are usually poorly developed and mostly ineffective swimmers, with initial dispersal largely a result of hydrodynamic processes [7]. However, many larvae develop the capability to orientate themselves within the water column, to swim against the current, and to control their trajectories [8,9]. Thus, while larvae often have the potential to move over vast distances during the pelagic phase, many also have the potential to remain close to their native reefs [7,10,11], leading to well-connected populations on scales of 0–30 km [12,13]. After this planktonic larval phase, which usually lasts from 1 to 64 weeks, larval fishes settle into benthic reef habitats. Settlement patterns are often species-specific, dependent on nocturnal, lunar, seasonal and inter-annual factors. Some factors appear to be particularly important, with the abundance of larvae settling to reefs often peaking during the night, new moon, summer, and La Niña periods [7,14,15].

While a substantial amount is known regarding larval transport, dispersal, aggregations, movement patterns, and their relationship to oceanographic features [7,16–19], relatively little research has examined how closely settlement patterns at one reef reflect settlement to neighbouring reefs [20–22]. Indeed, most research has sampled relative larval abundance using light traps [17,23,24] or towed nets [17,25,26], while studies that have examined connectivity, dispersal and recruitment patterns have mainly done so via *in situ* surveys of larvae in pelagic or reef environments [27–31], or through genetics analyses [19,32] and otolithometry [13,16,33]. However, deciphering precisely where and when these larvae settled out of the plankton is often either not possible, or prohibitively difficult and labour intensive, using these techniques.

Where the reef structure is appropriate, crest nets allow for the collection of settlement-stage larval assemblages, with almost no sampling bias due to larval behaviour (such as occurs with light traps) and almost no net avoidance due to the continuous current flow over the reef crest and the turbulence of the surf zone in front of the net [15,20–22,34,35]. Crest nets therefore provide an accurate measure of the abundance and assemblage of settlement-stage larvae (*i*.*e*. larval supply) on a given reef. The few previous studies that have examined geographic patterns of larval supply have identified minimal spatial variation between reef sites located within the same island, whether they were separated by only 200 m and had the same coastline orientation [20,22], or by more than 10 km with different coastline orientations [21]. However, these studies have generally only compared a limited number of locations and further work is needed to quantify the degree to which oceanographic features (*e*.*g*. currents and diffusion) and biological processes (*e*.*g*. spawning, swimming potential, behaviour, and mortality) do or do not synchronize the arrival of larvae across spatially separated reefs [5,18]. Identifying the degree of spatial and temporal consistency or variation in larval supply between disparate reefs could also provide important spatial information regarding larval patch sizes in the ocean [16,36]

In the present study, we used crest nets to measure the variation in larval supply at five reef sites located on two spatially-disparate French Polynesian Archipelagos (Moorea Island in the Society Archipelago, and Nengo-Nengo Atoll in the Tuamotu Archipelago) over a 41 day period. This study aimed to identify spatial and temporal variability in patterns of larval settlement (using abundance and assemblage of settlement-stage larvae as proxies) and the relationship between these patterns and external factors including lunar cycle, coastline orientation, and relative proximity.

## Materials and methods

### Ethics statement

This study was carried out in accordance with the guidelines of the French Polynesia committee for animal ethics, and the experiments were approved by the CRIOBE-IRCP animal ethics committee. This study did not involve endangered or protected species.

### Study sites

**Fig 1:**
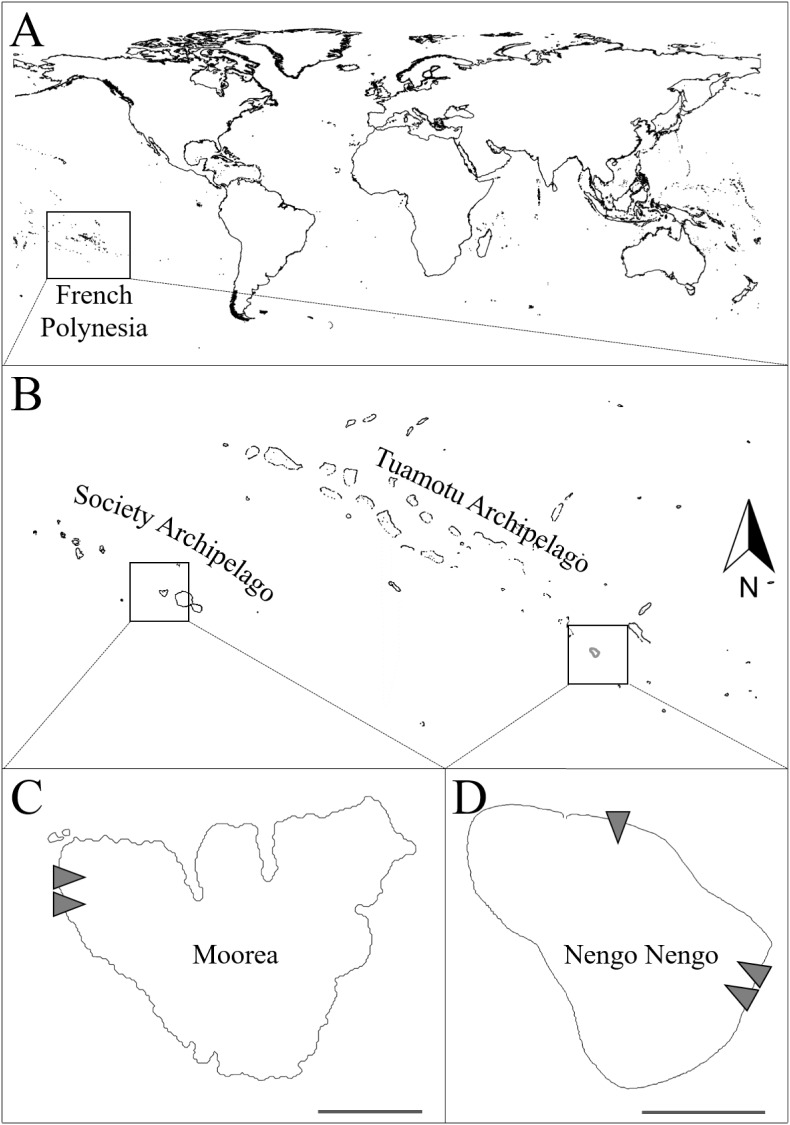
Study sites and crest net locations. (A) Location of French Polynesia within the Pacific Ocean. (B) Location of Moorea Island and Nengo-Nengo Atoll within French Polynesia. (C) Location of crest net sites. Grey triangles indicate crest net locations (M_W1 and M_W2 along the west coast of Moorea, NN_N on the north coast of Nengo-Nengo, and NN_SE1 and NN_SE2 along the south-east coast of Nengo-Nengo), as well as respective inflow orientations.

This study was conducted on the reef crests surrounding two islands in French Polynesia: along the west coast of Moorea Island (Tetaiuo sector: 17°31’7.38”S, 149°55’20.89”W) in the Society Archipelago, and along the north and south-east coasts of Nengo-Nengo Atoll (18°42’38.37”S, 141°49’6.20”W; 18°46’32.52”S, 141°45’43.57”W) in the Tuamotu Archipelago (Fig 1A and 1B). The distance between the Moorea and Nengo-Nengo sites is approximately 778 km. The supply of larval fishes at each site was recorded using crest nets: two at Moorea and three at Nengo-Nengo. At Moorea, both crest nets were set up along the west coast, spaced 100 m apart, and designated M_W1 and M_W2 (Moorea west crest net 1 and 2) (Fig 1C). At Nengo-Nengo, two crest nets were set up on the south-east coast, also spaced 100 m apart, and designated NN_SE1 and NN_SE2 (Nengo-Nengo south-east crest net 1 or 2), and one crest net was set up on the north coast, 7 km from the south-east nets, and designated NN_N (Nengo-Nengo north crest net) (Fig 1D). These five sites were chosen as they were: (i) accessible in most weather and tidal conditions, and (ii) had the correct reef crest structure for crest net installation. Replicating sites with the same coastline orientation at Moorea and Nengo-Nengo was not possible due to logistical difficulties.

### Sampling

The crest nets used to collect larval fishes followed a design used previously by Dufour and Galzin [15]. The body of each net was made of 2 mm mesh, allowing retention of all incoming larvae. Larvae entered the net via its rectangular mouth (width: 1.8 m; height: 1 m), oriented parallel to the reef crest, *i*.*e*. against the water flow and wave direction. For each net, the capture area was enlarged to a total of 5 m width through the addition of two hinged panels (width: 2m, height: 1 m) oriented at 40° on each side of the net entry. Using this method, all larvae entering the reef at this point were channelled down the net where they were collected in a cod-end for subsequent sampling. Cod-ends were only attached to the nets in the late afternoon to minimise the catch of debris during daylight hours when larval arrival onto the reef is minimal [15], and detached following sunrise to remove the larvae captured during the night. Following collection, all larval fishes were transferred to aquaria, where they were identified to the lowest taxonomic level possible following the key by Leis and Carson-Ewart [37], before being released. Larvae were collected from each of the five nets each morning between 10^th^ March and 14^th^ May 2011.

### Statistical analyses

All statistical analyses were conducted using the R-Cran project free software (http://www.rproject.org/, R-3.3.1). To determine if the total and daily abundance of settlement-stage larvae differed over the study period between Moorea Island and Nengo-Nengo Atoll, we performed Welch two-sample t-tests, when data met assumptions of normality (Shapiro test) and homogenous variance (Bartlett test). Wilcoxon rank sum tests were conducted when data did not meet these assumptions. We also determined if differences occurred between Moorea Island and Nengo-Nengo for the four most abundant species collected: *Acanthurus triostegus*, *Bothus mancus*, *Chromis viridis* and *Pristiapogon fraenatus*, by conducting the same statistical tests after normalizing species abundances to the abundance of all larvae captured at each site. We then compared within-site differences in daily abundance of settlement-stage larvae (*i*.*e*. among the five sites: both Moorea sites and the three Nengo-Nengo sites) using a Kruskal-Wallis test followed by a Kruskal-Nemenyi post hoc test.

To investigate if temporal variations in the supply of larval fishes (*i*.*e*. settlement patterns) were correlated with the lunar cycle, we performed cross correlation function (CCF) analyses using the *stats* package. These analyses compared the total and species-specific daily larval abundance at each site with the percentage of the moon that was illuminated (Table 1). Ranked daily larval abundance data were used as raw data did not meet the assumption of normality. CCF analyses resulted in a variety of “lags” (*i*.*e*. delay days), and their associated Spearman ρ, for which the daily larval abundance and the percentage of the moon that was illuminated were correlated. A negative “lag” indicates that variations in larval abundance (*e*.*g*. a peak or a minimum) occurred a certain number of days prior to variations in lunar illumination (*e*.*g*. full moon or new moon), while a positive “lag” indicates that variations in larval abundance occurred a certain number of days following variations in lunar illumination. For example, a “lag” value of “-2”, associated with a negative value Spearman ρ indicates that larval abundance and the percentage of the moon that is illuminated are correlated, with larval abundance peaking two days before minimum lunar illumination (*i*.*e*. new moon). Lags were sorted and removed if their associated Spearman ρ was < |0.40| (with an associated p-value > 0.01), and pooled if consecutive. For example, if lags “-2”,” -1”, “0”, “1” and “6” were sorted from a CCF analysis, with the highest correlation (highest absolute value of Spearman ρ) between the two series at lag = “-1”, results were presented the following way: -1 [-2:1;6], with Spearman ρ only given for the lag maximum (“-1” in this example) (see Tables 1–3).

**Table 1:** Larval settlement patterns in relation to lunar phases.

Species	CCF	lag	Spearman ρ
**All species**	M_W1	**1** [-2:2]	**-0.48** (**)
	M_W2	**1** [-1:2]	**-0.49** (**)
	NN_N	NS	NS
	NN_SE1	**-1** [-2:1]	**-0.58** (***)
	NN_SE2	**1** [-1:2]	**-0.57** (***)
***A*. *triostegus***	M_W1	**-1** [-2:1]	**-0.58** (***)
	M_W2	**0** [-2:2]	**-0.62** (***)
	NN_N	NS	NS
	NN_SE1	**-1** [-2:0]	**-0.50** (**)
	NN_SE2	NS	NS
***B*. *mancus***	M_W1	**2** [1:4]	**-0.53** (***)
	M_W2	NS	NS
	NN_N	NS	NS
	NN_SE1	**-1** [-2:1]	**-0.56** (***)
	NN_SE2	NS	NS
***C*. *viridis***	M_W1	**-8** [-9:-7]	**0.46** (**)
	M_W2	**-8** [-11:-8]	**0.42** (**)
		**-1** [-2:0]	**-0.47** (**)
	NN_N	**0** [-1:0]	**-0.43** (**)
	NN_SE1	**0** [-2:1]	**-0.54** (***)
	NN_SE2	**0** [-1:1]	**-0.47** (**)
***P*. *fraenatus***	M_W1	**-4**	**0.41** (**)
	M_W2	**-4**	**0.40** (**)
	NN_N	**-3** [-5:-2]	**-0.44** (**)
	NN_SE1	**-2** [-2:-1]	**-0.42** (**)
	NN_SE2	NS	NS

CCF analyses were performed with total and species-specific daily larval abundance as the first (X) variable, and percentage of the moon that was illuminated as the second (Y) variable. In the “lag” column, α [β:γ;δ] indicates that lags β to γ, as well as lag δ, are the lags of X for which the correlations between X and Y are significant, and that among all these lags α is the lag with the highest absolute Spearman ρ value. Spearman ρ coefficient is only indicated for lag α and *, ** and *** indicate that this correlation coefficient is significantly different from 0 with a p-value inferior to 0.05, 0.01 and 0.001 respectively. NS indicates that no significant correlation was sorted out the CCF analysis.

**Table 2:** Comparisons of larval settlement patterns between nets.

Species	CCF	lag	Spearman ρ
**All species**	M_W1 ~ M_W2	**0** [-2:2]	**0.96** (***)
	M_W1 ~ NN_N	NS	NS
	M_W1 ~ NN_SE1	NS	NS
	M-W1 ~ NN_SE2	NS	NS
	M_W2 ~ NN_N	NS	NS
	M_W2 ~ NN_SE1	NS	NS
	M_W2 ~ NN_SE2	NS	NS
	NN_N ~ NN_SE1	**0**	**0.42** (**)
	NN_N ~ NN_SE2	**0**	**0.52** (***)
	NN_SE1 ~ NN_SE2	**0** [-4:1]	**0.83** (***)
***A*. *triostegus***	M_W1 ~ M_W2	**1** [-2:2]	**0.79** (***)
	M_W1 ~ NN_N	**13**	**0.46** (**)
	M_W1 ~ NN_SE1	NS	NS
	M_W1 ~ NN_SE2	NS	NS
	M_W2 ~ NN_N	NS	NS
	M_W2 ~ NN_SE1	**3**	**0.43** (**)
	M_W2 ~ NN_SE2	NS	NS
	NN_N ~ NN_SE1	NS	NS
	NN_N ~ NN_SE2	NS	NS
	NN_SE1 ~ NN_SE2	NS	NS
***B*. *mancus***	M_W1 ~ M_W2	**0**	**0.49** (**)
	M_W1 ~ NN_N	NS	NS
	M_W1 ~ NN_SE1	NS	NS
	M_W1 ~ NN_SE2	NS	NS
	M_W2 ~ NN_N	NS	NS
	M_W2 ~ NN_SE1	NS	NS
	M_W2 ~ NN_SE2	NS	NS
	NN_N ~ NN_SE1	**0**	**0.67** (***)
	NN_N ~ NN_SE2	**0** [0;5]	**0.53** (***)
	NN_SE1 ~ NN_SE2	NS	NS
***C*. *viridis***	M_W1 ~ M_W2	**-6**	**-0.47** (**)
		**0** [-1:0]	**0.62** (***)
	M_W1 ~ NN_N	NS	NS
	M_W1 ~ NN_SE1	NS	NS
	M_W1 ~ NN_SE2	NS	NS
	M_W2 ~ NN_N	NS	NS
	M_W2 ~ NN_SE1	**1**	**0.40** (**)
	M_W2 ~ NN_SE2	NS	NS
	NN_N ~ NN_SE1	**0**	**0.81** (***)
	NN_N ~ NN_SE2	**0**	**0.73** (***)
	NN_SE1 ~ NN_SE2	**0** [-1:1;3]	**0.84** (***)
***P*. *fraenatus***	M_W1 ~ M_W2	**0** [0:1]	**0.73** (***)
	M_W1 ~ NN_N	NS	NS
	M_W1 ~ NN_SE1	**-3**	**-0.43** (**)
	M_W1 ~ NN_SE2	NS	NS
	M_W2 ~ NN_N	NS	NS
	M_W2 ~ NN_SE1	**-3**	**-0.41** (**)
	M_W2 ~ NN_SE2	NS	NS
	NN_N ~ NN_SE1	**0** [0:1]	**0.84** (***)
	NN_N ~ NN_SE2	**0** [-1:0]	**0.75** (***)
	NN_SE1 ~ NN_SE2	**0** [-1:0]	**0.73** (***)

Net1 ~ Net2 indicates that the CCF analyses was performed with total or species-specific daily larval abundance at site 1as the first (X) variable, and at site 2 as the second (Y) variable. In the “lag” column, α [β:γ;δ] indicates that lags β to γ, as well as lag δ, are the lags of X for which the correlations between X and Y are significant, and that among all these lags α is the lag with the highest absolute Spearman ρ value. Spearman ρ coefficient is only indicated for lag α and *, ** and *** indicate that this correlation coefficient is significantly different from 0 with a p-value inferior to 0.05, 0.01 and 0.001 respectively. NS indicates that no significant correlation was sorted out the CCF analysis.

**Table 3:** Comparisons of species versus total settlement patterns.

	CCF	lag	Spearman ρ
**M_NW1**	*A*. *triostegus*	**0** [-2:2]	**0.60** (***)
	*B*. *mancus*	NS	NS
	*C*. *viridis*	**-8**	**-0.45** (**)
		**-1** [-1;1]	**0.43** (**)
	*P*. *fraenatus*	NS	NS
**M_NW2**	*A*. *triostegus*	**-9**	**-0.41** (**)
		**-1** [-1:0]	**0.68** (***)
	*B*. *mancus*	NS	NS
	*C*. *viridis*	**0** [-2:1;7]	**0.44** (**)
	*P*. *fraenatus*	NS	NS
**NN_N**	*A*. *triostegus*	NS	NS
	*B*. *mancus*	NS	NS
	*C*. *viridis*	NS	NS
	*P*. *fraenatus*	**-1**	**0.43** (**)
**NN_SE1**	*A*. *triostegus*	**-1** [-1:6]	**0.67** (***)
	*B*. *mancus*	**0** [-1:1;4:5]	**0.66** (**)
	*C*. *viridis*	**0** [-2:3]	**0.73** (***)
	*P*. *fraenatus*	**0** [-3:1;4]	**0.71** (***)
**NN_SE2**	*A*. *triostegus*	NS	NS
	*B*. *mancus*	**-1** [-1:0]	**0.58** (***)
	*C*. *viridis*	**0** [-1:2]	**0.82** (***)
	*P*. *fraenatus*	NS	NS

CCF analyses were performed with species daily larval abundance at a site as the first (X) variable, and total daily larval abundance of the same site as the second (Y) variable. In the “lag” column, α [β:γ;δ] indicates that lags β to γ, as well as lag δ, are the lags of X for which the correlations between X and Y are significant, and that among all these lags α is the lag with the highest absolute Spearman ρ value. Spearman ρ coefficient is only indicated for lag α and *, ** and *** indicate that this correlation coefficient is significantly different from 0 with a p-value inferior to 0.05, 0.01 and 0.001 respectively. NS indicates that no significant correlation was sorted out the CCF analysis.

To investigate if variations in temporal supply of larval fishes were correlated among the five sites, we compared the total and species-specific daily larval abundances between each site (Table 2). To do so, we used CCF analyses in a similar way as described above. To investigate species-specific variations in temporal supply of fish larvae at each site (*i*.*e*. species-specific settlement patterns), we compared the daily larval abundances of each of the four dominant species with the total daily larval abundance of the associated site (Table 3). Again, we used CCF analyses in a similar way as described above.

## Results

**Fig 2:**
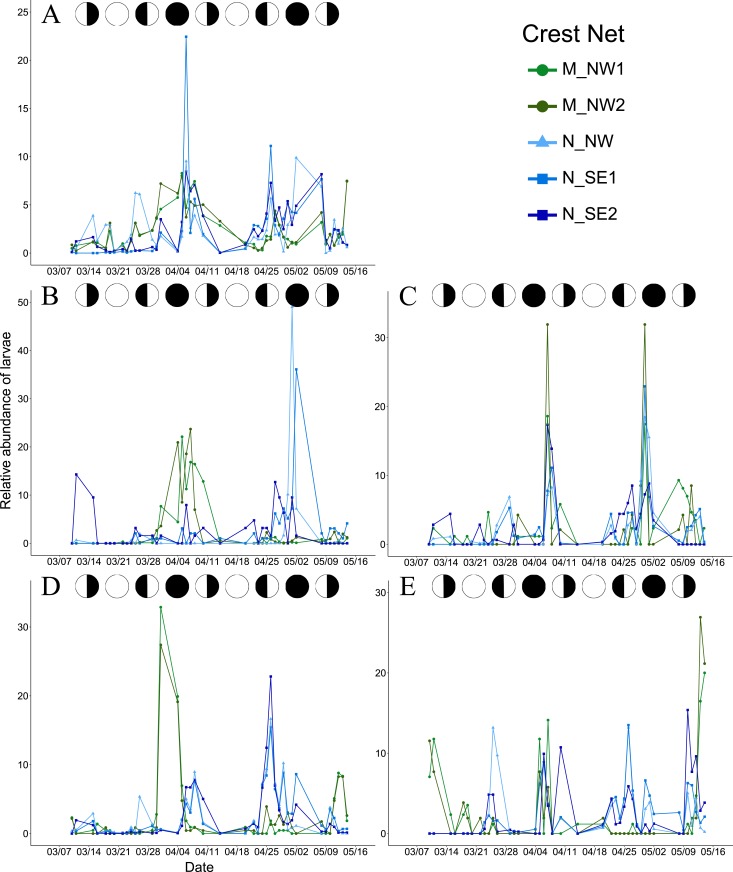
Relative abundances of larvae in each crest net. Relative abundance of (A) all larval fishes, (B) *Acanthurus triostegus* larvae, (C) *Bothus mancus* larvae, (D) *Chromis viridis* larvae, (E) *Pristiapogon fraenatus* larvae. Green and dark green lines indicate M_W1 and M_W2 nets respectively while light blue, blue, and dark blue lines indicate NN_N, NN_SE1 and NN_SE2. Circular points indicate a crest net with a westward orientation, triangular points indicate a northward orientation and square points indicate a south-eastward orientation. Black and white circles above each panel indicate the lunar phases: new moon (black circle), first quarter, full moon (white circle) and last quarter.

A total of 83,915 larval fishes belonging to 112 species were collected and identified from the five crest nets between March 10^th^ and May 14^th^, 2011. In Moorea, the two crest nets collected 4,892 and 4,225 larvae from 104 species (including 75 also found in the Nengo-Nengo catches), respectively. In Nengo-Nengo, 16,970 larval fishes were collected at the crest net located on the north coast, and the south-east coast crest nets collected 29 206 and 28 622 larval fishes from 95 species, respectively. Overall, the total larval abundance was 5 times higher in Nengo-Nengo than Moorea (Welch two sample t-test, t = -5.09, df = 2.03, p < 0.05), as was the daily larval abundance (Kruskal-Wallis χ^2^ = 42.13, df = 4, p<0.001). Within each location (Moorea and Nengo-Nengo), no significant difference in larval abundance was observed between nets (Kruskal-Wallis χ^2^ = 4, df = 4, p = 0.41). The relative abundance of larvae collected peaked at the beginning of April and in late April- early May (Fig 2A).

Among the 112 species captured, the four most abundant were: *Acanthurus triostegus* (0.2 to 23% of net catches), *Bothus mancus* (1.1 to 4.3%), *Chromis viridis* (4.4 to 28.3%) and *Pristiapogon fraenatus* (1.2 to 42.4%). The abundance of *A*. *triostegus* larvae was almost 34 times higher in Moorea compared to Nengo-Nengo (Welch two sample t-test, t = 10.78, df = 1.16, p < 0.05), while the abundance of *C*. *viridis* larvae was almost 5 times higher in Nengo-Nengo compared to Moorea (Welch two sample t-tests, t = -6.95, df = 2.14, p < 0.01). The abundance of *B*. *mancus* and *P*. *fraenatus* larvae were not significantly different between islands (Welch two sample t-test, t = -1.01, df = 2.42, p > 0.40, and Wilcoxon rank sum test, W = 0, p = 0.2). Except for *P*. *fraenatus*, the relative abundance of all species peaked at the beginning of April and May (Fig 2B–2F).

Peaks in total larval abundance occurred one day before or one day after the new moon, in both nets at Moorea and both south-east nets at Nengo-Nengo (Table 1). At the species level, the same temporal settlement pattern was observed for *A*. *triostegus* at M_W1, M_W2 and NN_SE1, *B*. *mancus* at M_W1 and NN_SE1, and *C*. *viridis* in all sites except M_W1 (Table 1). At M_W1 and M_W2, peaks in *C*. *viridis* larval abundance occurred 8 days before the full moon (Table 1). For *P*. *fraenatus*, peaks in larval abundance occurred 4 days before the full moon at M_W1 and M_W2, and several days before the new moon at NN_N and NN_SE1 (Table 1).

When considering all larvae, settlement patterns were similar between the two nets at Moorea and among the three nets at Nengo-Nengo (Table 2). At the species level, the larval settlement patterns of *A*. *triostegus*, *B*. *mancus*, *C*. *viridis* and *P*. *fraenatus* were similar between the two nets at Moorea (Table 2), while only the larval settlement patterns of *C*. *viridis* and *P*. *fraenatus* were consistent between the three nets at Nengo-Nengo (Table 2). While larval settlement patterns of *B*. *mancus* were not correlated between NN_SE1 and NN_SE2, both were found to be similar to NN_N individually (Table 2).

Finally, at M_W1 and M_W2, only *A*. *triostegus* and *C viridis* exhibited larval settlement patterns that were similar to the total pattern of their associated net (Table 3), while the larval abundance of all four key species peaked at the same time at NN_SE1 (Table 3). At NN_SE2 this was also the case for *B*. *mancus* and *C*. *viridis* (Table 3), while at NN_N, only *P*. *fraenatus* exhibited a larval settlement pattern that was correlated with the total pattern of its associated net, with a peak of abundance occurring one day before the peak of total abundance (Table 3).

## Discussion

Although spatial differences in larval abundance (total and daily) and assemblage structure were observed among the five monitored sites (Fig 2 and Table 2), temporal patterns of larval settlement were broadly similar in that peaks of larval supply occurred at the new moon at four of the five sites (Table 1). This finding conforms with previously observed patterns of larval settlement in this region as well as other reef areas [14,15,38]. This result also reveals that, even among sites with different coastline orientations (*i*.*e*. each submitted to different wind and swell inputs) and separated by almost 800 km, the lunar cycle had a greater influence than local habitat and hydrodynamic conditions in determining patterns of reef fish settlement.

At the species level, while an especially high abundance of settlement-stage *A*. *triostegus* in Moorea has been acknowledged previously along almost all coastlines [22,39], this is the first study to identify a high abundance of settlement-stage *C*. *viridis* (Pomacentridae), *P*. *fraenatus* (Apogonidae) and *B*. *mancus* (Bothidae) in an atoll in the Tuamotu Archipelago [38]. With regards to temporal settlement patterns, the abundance of *A*. *triostegus* larvae was correlated between both Moorea sites and one Nengo-Nengo site (NN_SE1), while the other dominant species exhibited distinct trends in larval abundance between the two islands (Tables 1 & 2). Patterns of *C*. *viridis* larval abundance were similar in both Moorea sites (Table 1), with peaks occurring approximately one week before the full moon; a period equivalent to that described by Dufour and Galzin [15], but different to that observed in the Nengo-Nengo sites, where larval abundance peaked around the new moon (Table 1).

Differences between Moorea and Nengo-Nengo with regards to both larval abundances and species-specific settlement patterns may be explained by the different coastline orientations of the crest nets at each location, if associated sampling sites were subject to different hydrographic pressures (*e*.*g*. currents, eddies, swells and winds). For example, both islands are located within the westward moving ‘Southern Equatorial Current’ [40], but Nengo-Nengo nets were oriented towards this current while those in Moorea were not. These differences in water influx could have affected larval supply at each location. However, Dufour et al. [21] demonstrated that patterns of larval settlement in Moorea can be homogeneous, even between sites separated by 8 to 12 km with different coastline orientations. This suggests that orientation to prevailing current does not play a central role in determining relative larval abundance. Although this study used a small number of crest net replicates, similar homogeneous patterns of larval settlement were observed at Nengo-Nengo, with larval abundances peaking simultaneously for three of the dominant species at all three sites (Table 2). Consequently, what is more likely is that differences in larval abundance between islands is a consequence of factors such as differences in the adult density [41], the relative size of each island’s lagoon (Nengo-Nengo lagoon area is 108 km^2^ against 61 km^2^ for Moorea), the topographic variation (high island vs. atoll environments), and the relative geographical proximity of other islands (several other atolls surround Nengo-Nengo, while Moorea is comparatively relatively isolated with only Tahiti high island and small Tetiaroa atoll nearby) [11,40]. In addition, as coral reef fish larvae use a variety of marine and land-based sensory cues to locate, differentiate between, and orientate towards settlement sites [42–45], the environmental differences that exist between the two islands could potentially affect their relative ability to attract settlement-stage larval fish. Moorea is a high island with rivers, bays, a developed coastline, twelve passes between the ocean and the lagoon that are distributed on all coastlines, and almost no tides [40]. Therefore, Moorea potentially emits sensory cues, such as waterborne chemicals from terrestrial vegetation, homogeneously and over limited spatial scales. In contrast, Nengo-Nengo is a remote, relatively undeveloped, atoll subject to a strong tidal influence and with only one pass [40]. Therefore, important cues, such as chemicals that indicate the location of the lagoon, are likely released heterogeneously (in the direction of the pass) and spread over a larger spatial area. Further work that compares patterns of larval settlement between spatially disparate sites that have the same coastline orientation will determine if an island’s environmental characteristics are more important than coastline orientation in determining the associated larval supply.

Spatial consistency in larval supply could also reflect the size of the larval patch in the ocean [16,17,22]. A substantial body of research suggests that the presence of certain oceanographic processes, such as wake eddies behind reefs, can promote the retention of larvae in patches around islands [12,13,18,46–49]. From these patches, larvae may then swim towards nearby reef habitats to settle following a pelagic waiting period [15], with the timing determined by broad-scale environmental conditions (*e*.*g*. the lunar phase), as well as local processes that affect recruitment (*e*.*g*. temporal patterns of spawning, narrow ranges in pelagic larval duration, variations in reef-associated noise, wind direction, or local dynamism of the water column) [7,50]. The results of this study do not provide definitive evidence for either the ‘large larval patch’ or ‘dilution of a smaller larval patch at the time of settlement’ hypotheses [16,17]. Nevertheless, the occurrence of synchronous larval settlement events with identical temporal patterns, even at the species level, at sites with different coastline orientations, were seen here at Nengo-Nengo and in a previous study at Moorea [21]. At sites with the same coastline orientation, previous studies have also identified consistency in temporal patterns of larval settlement among six sites separated by 200 m on the west coast of Moorea [22], and also between sites separated by 200 m on the west coast of Australia [20]. All these results point towards the presence of a large oceanic larval patch surrounding islands. Indeed it is unlikely that, in spatially disparate areas with different coastline orientations, larvae from small patches would exhibit precise synchronization with regards to settlement. Moreover, among multiple sites located within the same coastline, settlement of small patches would have resulted, at some sites, in an absence of settlers. However, this was not the case here or in other studies [20–22].

Examination of variation in larval supply in Moorea and Nengo-Nengo identified that larval abundance is generally higher during new moon periods, even between sites separated by up to 800 km. This result is in line with other studies, on other reef fish species, from the Caribbean [51,52], Mediterranean [53] and Pacific [15,20,21,38]. This study also highlights the value of crest nets as a mean for gathering accurate and unbiased estimations of larval supply in comparison to light traps (selective catch), otolithometry and *in situ* surveys (*i*.*e*. which reveal more recruitment than settlement patterns). Although we did observe differences between both islands, in particular regarding relative abundances at the species level (*i*.*e*. assemblage structure), there was a strong consistency in larval settlement patterns within each island, even among sites located along different coastlines. The reasons for this consistency are still unclear, but are likely linked to a variety of physical and biological processes, interacting with a large and diluted larval patch surrounding each island.

## Supporting information

S1 File Collected larval fishes and percentage of the moon that was illuminated. Raw data of the larval fishes that were collected during the study and data of the percentage of the moon that was illuminated.
(XLSX)
